# The Atypical Effective Connectivity of Right Temporoparietal Junction in Autism Spectrum Disorder: A Multi-Site Study

**DOI:** 10.3389/fnins.2022.927556

**Published:** 2022-07-18

**Authors:** Zeqi Hao, Yuyu Shi, Lina Huang, Jiawei Sun, Mengting Li, Yanyan Gao, Jing Li, Qianqian Wang, Linlin Zhan, Qingguo Ding, Xize Jia, Huayun Li

**Affiliations:** ^1^School of Teacher Education, Zhejiang Normal University, Jinhua, China; ^2^Key Laboratory of Intelligent Education Technology and Application, Zhejiang Normal University, Jinhua, China; ^3^Department of Radiology, Changshu No. 2 People's Hospital, The Affiliated Changshu Hospital of Xuzhou Medical University, Changshu, China; ^4^School of Information and Electronics Technology, Jiamusi University, Jiamusi, China; ^5^School of Western Languages, Heilongjiang University, Harbin, China

**Keywords:** autism spectrum disorder, temporoparietal junction, Granger causality analysis, multi-site, image-based meta-analysis

## Abstract

Social function impairment is the core deficit of autism spectrum disorder (ASD). Although many studies have investigated ASD through a variety of neuroimaging tools, its brain mechanism of social function remains unclear due to its complex and heterogeneous symptoms. The present study aimed to use resting-state functional magnetic imaging data to explore effective connectivity between the right temporoparietal junction (RTPJ), one of the key brain regions associated with social impairment of individuals with ASD, and the whole brain to further deepen our understanding of the neuropathological mechanism of ASD. This study involved 1,454 participants from 23 sites from the Autism Brain Imaging Data Exchange (ABIDE) public dataset, which included 618 individuals with ASD and 836 with typical development (TD). First, a voxel-wise Granger causality analysis (GCA) was conducted with the RTPJ selected as the region of interest (ROI) to investigate the differences in effective connectivity between the ASD and TD groups in every site. Next, to obtain further accurate and representative results, an image-based meta-analysis was implemented to further analyze the GCA results of each site. Our results demonstrated abnormal causal connectivity between the RTPJ and the widely distributed brain regions and that the connectivity has been associated with social impairment in individuals with ASD. The current study could help to further elucidate the pathological mechanisms of ASD and provides a new perspective for future research.

## Introduction

Autism spectrum disorder (ASD) is a neurodevelopmental disorder with impaired social function as its core deficit (Lord et al., 2018), and the global prevalence is estimated to be 1–1.5% (Baxter et al., 2015). Social impairment can affect access to higher education, employment, independent living, and intimate relationships (Poon and Sidhu, 2017) and lead to a lower overall quality of life for individuals with ASD (Howlin and Magiati, 2017). However, the limited understanding of the pathological mechanism leads to the limited therapeutic effect of ASD (Sharma et al., 2018; Xu et al., 2019; Wood et al., 2020; Hickman et al., 2022; Lord et al., 2022). Researchers have utilized advanced brain imaging techniques and analytic methods to elucidate the biological underpinnings of ASD to facilitate early screening and treatment options for individuals with ASD to improve their quality of life (Wang et al., 2022; Zhao et al., 2022).

Previous studies demonstrated that the right temporoparietal junction (RTPJ) not only played an important role in the “social brain” of ASD (Pelphrey et al., 2011; Müeller and Fishman, 2018) but is also involved in higher-order social cognition such as processing the intentions and opinions of others (Wang et al., 2020b), reciprocating face-to-face social interactions (Tang et al., 2016), processing social faces (Kret et al., 2011), and reciprocating interpersonal empathy (Patel et al., 2019; Canigueral et al., 2021). However, the RTPJ cannot perform these social functions alone and needs to cooperate with other brain regions. Examination of the correlation of intrinsic brain activity between the RTPJ and other brain regions can help us to further explore the role played by the RTPJ in the pathophysiological mechanisms of ASD. The resting-state functional connectivity (rs-FC), a commonly used analytic method to provide information on intrinsic synchronized activity in the brain (Fishman et al., 2018; Gotts et al., 2019), demonstrated that the RTPJ had hyperconnectivity with the fusiform gyrus, which is associated with social difficulty in ASD (Chien et al., 2015). Hoffmann et al. conducted a study on self-other distinction and found that the functional connectivity between the RTPJ and the prefrontal cortex (PFC) was reduced in individuals with ASD (Hoffmann et al., 2016). Although previous studies demonstrated that social impairment in individuals with ASD was associated with altered FC in the RTPJ, it is difficult to characterize causal influences between regions and within circuits using only this method (Wicker et al., 2008; Wang et al., 2022). In contrast, effective connectivity can reflect the influence exerted by one neuron system on another in a particular direction and provide information closely related to the causal processes that operate in brain function (Friston, 1994; Wei et al., 2020). Specifically, by comparing the effective connectivity differences between the ASD group and the typical development (TD) group, it could provide information about how impaired brain regions affected other brain regions in ASD (Rolls et al., 2020). This could help researchers better understand the pathophysiological mechanisms of ASD.

Granger causality analysis (GCA) is an exploratory data-driven approach (Wang et al., 2022), which can be used to measure the direction of information flow between brain regions and estimate resting-state directional brain networks (Goebel et al., 2003; Roebroeck et al., 2005; Uddin et al., 2011). Previous studies demonstrated the important role of GCA in exploring the underlying pathological mechanisms of social impairment in individuals with ASD (Chen et al., 2016; Li et al., 2021), which were used to investigate abnormal excitatory or inhibitory directional connectivity between brain regions in individuals with ASD and could identify their underlying social impairment (Liao et al., 2011; Uddin et al., 2011). However, previous GCA studies focused on the study of neural circuits during specific social tasks in ASD, and the mechanisms of spontaneous neural activity interactions between brain regions in the absence of an explicit task need to be further explored (Li et al., 2021). Furthermore, the blood oxygen level-dependent (BOLD) signal measured by resting-state functional MRI (rs-fMRI) is convoluted by underlying neural activity and hemodynamic response function (HRF). Considering that HRF varies in different brain regions due to many neural and non-neural factors (Handwerker et al., 2012; Badillo et al., 2013), the assumption that HRF is homogeneous in the brain may subject the interpretation of results to HRF confounding effects (Rangaprakash et al., 2018). Therefore, we used a blind deconvolution technique to extract the region-specific HRF and deconvolve the BOLD signal into a neural signal (Wu et al., 2013, 2021), which could minimize the confound of non-neural HRF inherent in the BOLD signal (Wu and Marinazzo, 2016).

Previous rs-fMRI studies on ASD commonly used a small sample of participants from a single site, which led to limited statistical power (Van Horn and Toga, 2009). Nowadays, multisite data are used to improve the statistical power by increasing the total sample size of the study (Button et al., 2013; Yu et al., 2018). Besides, multisite data could enroll more representative samples which benefit from generalizing the study results to a diverse population (McGonigle, 2012; Dansereau et al., 2017). Therefore, we conducted our research based on the Autism Brain Imaging Data Exchange (ABIDE), a large sample of a multisite public dataset (http://fcon_1000.projects.nitrc.org/indi/abide/), to improve the reproducibility and statistical power of the results. It includes two large-scale collections: ABIDE I and ABIDE II, each of which is composed of independent functional and structural brain imaging data collected from more than 24 different laboratories worldwide (Di Martino et al., 2014, 2017). Although the large sample of multisite dataset addresses the impact of a small sample on research, there are significant challenges that remain in the analysis of multisite data, since fMRI data from different sites may contain scanner and site variability, which may lead to inconsistent results and low reliability (Friedman et al., 2008; Abraham et al., 2017). To solve this problem, we used anisotropic effect-size signed differential mapping (AES-SDM) to conduct an image-based meta-analysis using the uncorrected statistical parametric map (Radua et al., 2012, 2014), which is a powerful method that can solve the issues caused by heterogeneity among different sites and is useful for distinguishing spurious results from replicable findings (Fox et al., 2014; Müller et al., 2018).

To address the above questions, we first conducted GCA using RTPJ as the region of interest to investigate the differences in effective connectivity between individuals with ASD and TD. Then, an image-based meta-analysis was applied to identify the consistent brain regions that showed a significant difference between the two groups. We hypothesized that individuals with ASD had atypical effective connectivity between the RTPJ and brain regions related to a social function, such as the fusiform and PFC. We hope that the present study could help us further explore the brain mechanisms of social impairment in individuals with ASD and would help to develop the treatment in the future.

## Methods

### Participants

The experimental data of this study were obtained from the ABIDE dataset (ABIDE I and ABIDE II), which included multiple neuroimaging data sites. Before data collection, all sites were required to confirm that their local institutional review board (IRB) or ethics committee approved both the initial collection and retrospective sharing of a fully anonymized version of the datasets (Di Martino et al., 2014, 2017). A total of 23 sites were included in this study. These sites were further divided into 30 groups according to the number of slices and time points. The participants were divided into the ASD and TD groups, which contained 618 and 836 participants, respectively. The inclusion criteria included (1) participants with corresponding T1 image; (2) participants with lower head movement (translation or rotation <3 mm or 3°); (3) images with good normalization effect [we provided the normalization pictures in the website (http://restfmri.net/ABIDE-RTPJ-FC.zip)]; and (4) participants with right-handedness. The demographic information is shown in Table 1, and detailed information on the inclusion criteria is provided in the Supplementary Table S1. The scan information, diagnostic tools, and ethics statement of all the participants can be found at http://fcon_1000.projects.nitrc.org/indi/abide/.

**Table 1:** Demographic characteristics of ASD and TD.

**Site_ID**	**ASD (*N* = 618)**			**TD (*N* = 836)**				
	***N***	**Age (M ±SD)**	**Gender (male/female)**	***N***	**Age (M ±SD)**	**Gender (male/female)**	**Age *P-*value**	**Gender *P-*value**
001_BNI	22	35.32 ± 15.43	22/0	21	36.95 ± 15.20	21/0	0.728	—
002_Caltech	4	25.00 ± 8.48	4/0	10	28.03 ± 12.23	6/4	0.662	0.134
003_EMC	13	8.37 ± 1.16	10/3	14	8.02 ± 0.80	11/3	0.363	0.918
004_ETH	6	21.07 ± 4.10	6/0	19	23.37 ± 4.78	19/0	0.300	—
005_GU	22	10.93 ± 1.56	20/2	31	10.59 ± 1.87	15/16	0.490	0.001^b^
006_IP	15	24.53 ± 11.42	7/8	17	17.10 ± 7.86	9/8	0.038^a^	0.723
007_IU	12	25.92 ± 11.83	9/3	15	23.80 ± 5.23	11/4	0.573	0.922
008_KKI_1	13	9.75 ± 1.57	9/4	28	10.34 ± 1.07	16/12	0.163	0.460
009_KKI_2	41	10.57 ± 1.46	30/11	108	10.31 ± 1.19	66/42	0.254	0.170
010_Leuven	24	18.05 ± 5.20	22/2	27	18.87 ± 5.21	23/4	0.581	0.473
011_MaxMun_1	11	36.09 ± 11.75	8/3	12	33.25 ± 8.97	8/4	0.519	0.752
012_MaxMun_2	2	15.00 ± 9.90	2/0	14	26.43 ± 3.74	14/0	0.346	—
013_MaxMun_3	8	19.25 ± 13.30	8/0	2	9.00 ± 2.83	2/0	0.329	—
014_NYU	67	14.37 ± 7.92	55/12	98	14.97 ± 6.41	78/20	0.594	0.690
015_OHSU_1	13	11.66 ± 2.25	13/0	13	10.21 ± 1.09	13/0	0.051	—
016_OHSU_2	31	11.71 ± 2.30	25/6	51	10.39 ± 1.70	26/25	0.004^a^	0.007^b^
017_Olin1	14	17.21 ± 3.77	12/2	11	17.55 ± 3.96	9/2	0.833	0.792
018_Olin2	10	21.50 ± 3.95	10/0	29	24.34 ± 3.69	16/13	0.046^a^	0.010^b^
019_Pitt	22	18.94 ± 7.17	19/3	23	19.45 ± 6.60	20/3	0.805	0.953
020_SBL	9	35.00 ± 7.43	9/0	10	35.50 ± 4.86	10/0	0.863	—
021_SDSU	37	13.77 ± 2.96	31/6	41	13.81 ± 2.35	34/7	0.948	0.919
022_Stanford1	17	9.97 ± 1.62	14/3	16	10.11 ± 1.66	12/4	0.806	0.606
023_Stanford2	17	11.03 ± 1.23	16/1	18	10.99 ± 1.36	16/2	0.935	0.581
024_Trinity1	20	17.05 ± 2.68	20/0	23	17.48 ± 3.66	23/0	0.663	—
025_Trinity2	13	14.29 ± 3.60	13/0	18	16.28 ± 2.79	18/0	0.093	—
026_UMIA	10	10.29 ± 1.85	8/2	8	9.56 ± 1.82	6/2	0.415	0.800
027_UCD	16	14.81 ± 1.92	12/4	13	14.93 ± 1.71	10/3	0.866	0.904
028_UCLA	49	12.88 ± 2.40	45/4	47	12.15 ± 2.47	37/10	0.143	0.069
029_UM	37	13.56 ± 2.43	30/7	59	15.09 ± 3.61	44/15	0.026^a^	0.461
030_USM	43	22.90 ± 8.20	43/0	40	22.68 ± 6.63	38/2	0.892	0.138

*N, number; M, mean; SD, standard deviation*.

a *The P-value was calculated by the two sample t-test*.

b *The P-value was calculated by two-tailed Pearson chi-square t-test*.

*There was no Gender P in 001_BNI, 004_ETH, 011_MaxMun_1, 012_MaxMun_2, 015_OHSU_1, 020_SBL, 024_Trinity1 and 025_Trinity2 due to no female participants in these sites*.

### Resting-State fMRI Data Preprocessing

Resting-State fMRI Data Analysis Toolkit plus (RESTplus V1.24, http://restfmri.net/forum/restplus; Jia et al., 2019), based on Statistical Parametric Mapping (SPM12, http://www.fil.ion.ucl.ac.uk/spm), was used to preprocess the data on MATLAB 2017b (https://www.mathworks.cn/products/matlab.html). The first 10 time points were removed to promote equilibrium magnetization and allow the participants to adapt to the MRI environment. Subsequently, slice-timing was used to correct the acquisition time of differences between slices, and realignment was performed to adjust the time series of images to make every image at the same position (Yan and Zang, 2010). Next, the realigned images were spatially normalized to the Montreal Neurological Institute (MNI) space using the new segment method and resampled to 3 × 3 × 3 mm^3^ for the intersubject comparison to be feasible. Then, we used a 6 mm full-width half-maximum (FWHM) isotropic Gaussian kernel to perform spatial smoothing to reduce spatial noise (Wei et al., 2015; Shi et al., 2019; Dong et al., 2020; de la Cruz et al., 2021). Moreover, we regressed Friston 24 head motion parameters and signals of white matter and cerebrospinal fluid as nuisance signals to further reduce the effects of head motion (Friston et al., 1996) and non-neuronal BOLD fluctuations (Fox et al., 2005). Finally, the functional images were detrended to reduce systematic increase or decrease in the signal with time caused by long-term physiological shifts, movement-related noise remaining after realignment, or instrumental instability (Turner, 1997; Lowe and Russell, 1999). We did not perform band-pass filtering because previous studies considered that the model order in GCA was low (Hamilton et al., 2011; Liao et al., 2011; Wu et al., 2013).

### Blind Deconvolution Procedure

To minimize confounding effects introduced by the hemodynamic response function (HRF), we used Resting-State Hemodynamic Response Function Retrieval and Deconvolution (RS-HRF, https://www.nitrc.org/projects/rshrf) to extract the region-specific HRF and deconvolve the observed BOLD signal into the real neural signal (Wu et al., 2013, 2021).

### Granger Causality Analysis

After preprocessing, we used a voxel-wise GCA with the RTPJ as the region of interest to study the effective connectivity of brain regions using RESTplus. The MNI coordinates of RTPJ were centered at *x* = 54, *y* = −52, and *z* = 26, with a radius of 10 mm (Krall et al., 2015), and its time series was defined as seed time series X, while time series Y represented the time series of all the other voxels in the whole brain. In the GCA, if the future value of X or Y generated from the joint prediction of past values of the time series X and Y was better than that of the single prediction of X or Y, it was considered that there was a granger causality between X to Y or Y to X (Granger, 1969). *F*_x → *y*_ represented the ability to send information from the seed ROI to the whole brain and *F*_y → *x*_ represented the ability of the seed ROI to receive information from the whole brain. Next, we converted the coefficient-based *F*-to-*Z* score, namely, *Z*_x → *y*_ and *Z*_y → *x*_ (Zang et al., 2012).

### Statistical Analysis

For demographic and behavioral data, the statistical analyses were performed by Statistical Product and Service Solutions version (SPSS 20.0, IBM, Armonk, NY, USA). Continuous variables were compared using two-sample *t*-tests, and categorical variables were compared using chi-squared tests. Group differences were considered significant if *p*-value is < 0.05.

For the statistical analysis of the GCA, we used RESTplus to conduct a two-sample *t*-test between the ASD and TD groups with age, gender, and mean frame displacement (FD; Jenkinson et al., 2002) as covariates. To make the subsequent image-based meta-analysis to integrate all statistical information from the whole brain and improve the statistical power (Salimi-Khorshidi et al., 2009; Müller et al., 2018; Jia et al., 2021), we did not conduct multiple comparison correction for the statistical analysis results of GCA and shared the uncorrected statistical parametric maps (http://restfmri.net/ABIDE-RTPJ-FC.zip).

Then, AES-SDM Software V5.15 (https://www.sdmproject.com) was used to conduct a meta-analysis on the original uncorrected statistical parametric maps of the GCA results calculated from the ABIDE dataset (Radua et al., 2012, 2014). AES-SDM calculated the effect-size maps and effect-size variance maps by integrating the statistical parametric map and the differences in effect sizes between the groups. In our study, we first set the gray matter correlation template with the recommended full anisotropy = 1.0. Subsequently, to better balance the sensitivity and specificity of AES-SDM and to improve statistical stability, FWHM = 20 mm and 20 randomizations were applied to preprocess the original uncorrected *t*-maps of the GCA of all the included sites (Radua et al., 2012, 2014). Next, we performed a mean analysis, i.e., the main meta-analysis method. The main threshold of a *p*-value < 0.0005 (Chan and Han, 2020) and an additional *z*-based threshold of *z* > 1 were adopted to reduce the possibility of false-positive results. Then, the recommended extent threshold of 10 voxels was applied to exclude smaller clusters (Radua et al., 2012). Finally, we created a mask to extract the values of the areas with significant differences to further present the results.

## Results

### Demographic and Clinical Information

Initially, a total of 2,168 participants from ABIDE I and ABIDE II were included in our study. Among these, 714 participants were excluded for the following reasons: 30 participants were deleted for missing functional or structural images, 213 for poor normalization effect, 228 for excessive head motion (translation or rotation more than 3 mm or 3°), 188 not being right-handed, and four sites (55 participants) were excluded due to no TD participants. Finally, 618 individuals with ASD and 836 TD participants were included. Demographic information is shown in Table 1.

### GCA Meta-Analysis Results

#### Seed to the Whole Brain GCA in Meta-Analysis

**Figure 1:**
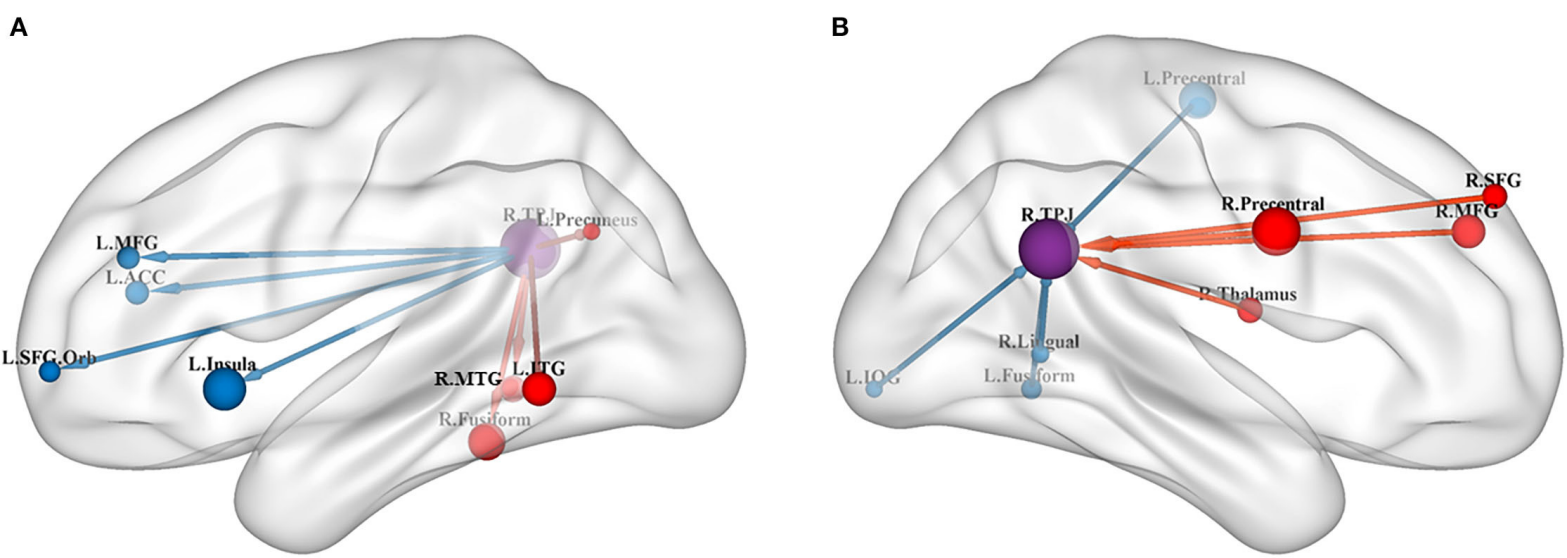
**(A)** Results of GCA from the RTPJ to the whole brain. **(B)** Results of GCA from the whole brain to the RTPJ. The red and blue lines represent increased or decreased effective connectivity between brain regions, respectively. The arrows demonstrated the direction of information transferred between brain regions. RTPJ, right temporoparietal junction; L.Fusiform, left fusiform; R.Fusiform, right fusiform; L.ITG, left inferior temporal gyrus; R.MTG, right middle temporal gyrus; L.Precuneus, left precuneus; L.Precentral, left precentral gyrus; R.Precentral, right precentral gyrus; L.Insula, left insula; L.ACC, left anterior cingulate cortex; L.MFG, left middle frontal gyrus; R.MFG, right middle frontal gyrus; L.SFG.Orb_L, left superior frontal gyrus, orbital part; R.SFG, right superior frontal gyrus; R.Thalamus, right thalamus; R.Lingual, right lingual; L.IOG, left inferior occipital gyrus.

The results of the meta-analysis of the GCA showed that, compared with the TD group, the GCA values of the ASD group from the RTPJ to the right fusiform, the left inferior temporal gyrus (ITG), the right middle temporal gyrus (MTG), and the left precuneus were higher. However, the left insula, the left anterior cingulate cortex (ACC), the left middle frontal gyrus (MFG), and the left superior frontal gyrus (SFG) were lower than those of the TD group (*p* < 0.0005, Table 2, Figure 1A).

**Table 2:** The GCA results from seed to the whole brain.

*F*_*x→y*_	**Anatomical label**	**BA**	**Number of voxels**	**Peak MNI coordinates [x, y, z]**	**Peak SDM-z value**
ASD > HC					
	Fusiform_R	37	21	32, −42, −18	3.71
	Temporal_Inf_L	37	32	−60, −54, −6	3.58
	Temporal_Mid_R	37	59	62, −48, −6	3.34
	Precuneus_L	NA	18	2, −66, 30	3.02
ASD < HC					
	Insula_L	47	98	−40, 18, −6	−3.90
	Cingulum_Ant_L	24	12	0, 38, 16	−3.24
	Frontal_Mid_L	46	10	−26, 40, 24	−3.21
	Frontal_Sup_Orb_L	11	12	−30, 58, −2	−3.16

*A cluster located in the cerebellum is not reported due to the incomplete partial site scan*.

*Fusiform_R, right fusiform; Temporal_Inf_L, left inferior temporal gyrus; Temporal_Mid_R, right middle temporal gyrus; Precuneus_L, left precuneus; Insula_L, left insula; Cingulum_Ant_L, left anterior cingulate cortex; Frontal_Mid_L, left middle frontal gyrus; Frontal_Sup_Orb_L, left superior frontal gyrus, orbital part; BA, Brodmann area; MNI, Montreal Neurological Institute; ASD, autism spectrum disorder; HC, healthy control; NA, not available*.

#### The Whole Brain to Seed GCA in Meta-Analysis

Compared with the TD group, the results of the meta-analysis of the GCA showed that the GCA values of the ASD group from the right precentral gyrus, the right MFG, the right thalamus, and the right SFG to the RTPJ were significantly higher than those of the TD group. However, the GCA values from the left precentral gyrus, the left fusiform, the right lingual gyrus, and the left inferior occipital gyrus (IOG) to the RTPJ were significantly lower than those of the TD group (*p* < 0.0005, Table 3, Figure 1B).

**Table 3:** The GCA results from the whole brain to seed.

*F*_*y→x*_	**Anatomical label**	**BA**	**Number of voxels**	**Peak MNI coordinates [x, y, z]**	**Peak SDM-z value**
ASD > HC					
	Precentral_R	6	16	54, 0, 30	4.00
	Frontal_Mid_R	46	15	22, 44, 30	3.39
	Thalamus_R	NA	12	12, −6, 12	3.05
	Frontal_Sup_R	9	11	18, 50, 38	3.03
ASD < HC					
	Precentral_L	4	12	−42, −18, 60	−3.54
	Fusiform_L	37	17	−30, −56, −6	−2.85
	Lingual_R	18	14	12, −54, 2	−2.75
	Occipital_Inf_L	18	13	−18, −92, −6	−2.71

*A cluster located in the cerebellum is not reported due to an incomplete partial site scan*.

*Precentral_R, right precentral gyrus; Frontal_Mid_R, right middle frontal gyrus; Thalamus_R, right thalamus; Frontal_Sup_R, right superior frontal gyrus; Precentral_L, left precentral gyrus; Fusiform_L, left fusiform; Lingual_R, right lingual; Occipital_Inf_L, left inferior occipital gyrus; BA, Brodmann area; MNI, Montreal Neurological Institute; NA, not available*.

## Discussion

In the current study, we conducted an image-based mate-analysis to explore the GCA alterations of ASD with the RTPJ as the ROI. Our results demonstrated that the atypical effective connectivity of the RTPJ in individuals with ASD was widely distributed, mainly in the fusiform, the insula, and the PFC. These brain regions play essential roles in face processing, self-awareness, and understanding others and are associated with social impairment in individuals with ASD. This suggested that the RTPJ is a multi-functional brain region that is extensively involved in multiple functions related to society. From the perspective of the direction of interaction between RTPJ and the above brain regions, we could deepen our understanding of the brain mechanisms of social impairment in ASD.

The fusiform, lingual gyrus, and IOG are all important components of the ventral occipitotemporal cortex (VOTC) and are jointly involved in the processing of visual stimuli, especially in relation to face recognition of ASD (Humphreys et al., 2008; Price and Devlin, 2011; Domes et al., 2013; Mamashli et al., 2021). Face recognition is a necessary social skill in social activities such as peer interaction, while its deficit is considered a core deficit in individuals with ASD (Griffin et al., 2021). The fusiform is most closely related to face recognition, and it is also one of the brain regions of the “social brain” (Frith and Frith, 2010). Previous studies showed increased functional connectivity of the RTPJ with the fusiform and lingual gyrus in ASD (Chien et al., 2015). Besides, the MTG is also associated with face processing disorder in ASD (van Veluw and Chance, 2014; O'Hearn et al., 2020; Liu et al., 2021). Our results supported these results and further revealed the direction of the connectivity. Specifically, the ASD group showed higher effective connectivity from the RTPJ to the fusiform and MTG, but the effective connectivity from the fusiform, the lingual gyrus, and the IOG to the RTPJ was lower than TD. Notably, the neural circuit between the RTPJ and the fusiform might reflect that impaired fusiform function could affect its ability to transmit face processing information to the RTPJ, a brain region responsible for integrating external input stimuli, and in turn, the RTPJ would like to send more information to the fusiform. This might suggest interference in the processing of important inputs related to social cognition and is associated with social impairment with ASD.

Self-awareness is a pivotal component of conscious experience and conscious self-regulation of behavior (Lou, 2012). Some studies demonstrated that individuals with ASD had impaired self-awareness (Verhoeven et al., 2012), performed stronger self-experience, and had clearer self-boundaries (Crespi and Dinsdale, 2019; Mul et al., 2019). Self-awareness includes physical self-awareness and mental self-awareness. The insula was an important brain region that constituted the core of the physical self-awareness (Wiebking et al., 2014, 2015) and was responsible for primary (objective) interoceptive signals re-represented to higher (subjective) sensory states through the integration of information with other brain regions (Craig, 2009; Wang et al., 2020a). Previous studies demonstrated that the TPJ is connected to the anterior insula *via* the middle longitudinal fasciculus and extreme capsule (Saur et al., 2008). TPJ collaborated with the insula to integrate the multiple sensory and sensorimotor signals, thus constituting a coherent physical self-awareness (Park and Blanke, 2019). Our results suggested that ASD had lower effective connectivity from the left insula to the RTPJ, which is consistent with previous research. It was found that disrupted connectivity between the TPJ and the insula might cause individuals to lose awareness of the contralesional half of the body (Committeri et al., 2006). We further provided directional information for this disruption of connectivity, and this “personal neglect” might be associated with insufficient information received by the TPJ from the insula. The insula is not only involved in the processing of the physical self-awareness but also in the processing of the mental self-awareness (Qin et al., 2020) together with brain regions of the default mode network, such as the precuneus (van Veluw and Chance, 2014) and the ACC (Kana et al., 2017). Our study found that effective connectivity from the RTPJ to the left precuneus was increased and effective connectivity from the RTPJ to the left insula and the left ACC was decreased in the ASD group. Although the insula, the ACC, and the precuneus were all related to impaired self-processing in ASD, their underlying mechanisms are different and future studies should further investigate the differences in the interaction between brain regions.

Furthermore, previous studies found that the ITG (Apps et al., 2012) and the precentral gyrus (Olivé et al., 2015; Rabellino et al., 2018) were also involved in self-awareness processing. However, few have investigated functional connectivity or effective connectivity between these brain regions and RTPJ. Our results revealed the effective connectivity from the RTPJ to the left ITG was higher in the ASD group. The effective connectivity from the right precentral gyrus to RTPJ was higher but was lower from the left precentral gyrus to the RTPJ. This might be affected by the abnormality of the sensorimotor cortex in ASD and is related to impaired self-awareness and social functions.

The PFC plays an important role in the social understanding of others, where the connectivity with the TPJ is primarily related to understanding the mental state of others (Andrews-Hanna et al., 2010; Libero et al., 2014). Previous studies showed that functional connectivity between the PFC and the TPJ decreased when individuals with ASD performed the social task (Burnett and Blakemore, 2009; Baumgartner et al., 2012). Our results demonstrated that the effective connectivity of the ASD group was lower than that of the TD group from the RTPJ to the left MFG and the left SFG but higher than that from the right MFG and the right SFG to the RTPJ. This might be related to the different functions between the TPJ and PFC in understanding the mental states of others. Previous research found that the PFC is associated with internal focusing processes that understand the content of others' thoughts, whereas the TPJ is associated with external focusing processes (Lieberman, 2007). Therefore, the atypical neural circuit between the RTPJ and the PFC suggests that individuals with ASD have difficulty integrating internal states and external features of others and are related to ASD's impairment in distinguishing between self and others. In addition to PFC, we also found that the effective connectivity from the right thalamus to the RTPJ was higher in the ASD group. This is consistent with previous studies, which suggested that enhanced functional connectivity between the TPJ and the thalamus in individuals with ASD reflects increased stress in social cognitive processes and affects the social perceptual performance of the TPJ (Kana et al., 2014; Woodward et al., 2017).

Previous studies suggested that GCA may be affected by HRF confounding effects due to variability in timing, amplitude, shape, and latency of HRF in different brain regions and participants (Miezin et al., 2000; Handwerker et al., 2012; Badillo et al., 2013). To overcome this problem, we used a blind deconvolution technique developed by Wu et al. to eliminate the effect of HRF and deconvolute BOLD signals into neural signals (Wu et al., 2013, 2021). The effectiveness of this technique has been recognized in studies of loss of consciousness induced by anesthesia and pathology (Wu et al., 2019), chronic pain without explicit onset (Wu and Marinazzo, 2015), and heart rate variability (Wu and Marinazzo, 2016).

There are some limitations to our study. First, we only chose the RTPJ as the region of interest since the TPJ on the right hemisphere was related to attention, empathy, and other social functions. However, this may ignore some important information about the TPJ on the left side. Second, cross-sectional group data from the public dataset of the present study did not allow us to assess the dynamic change of the GCA; therefore, future longitudinal studies are needed. Third, the lack of behavioral measures in the current study led to a limited interpretation of the fMRI results, which should be combined with behavioral measures in future studies to better interpret the fMRI results. Finally, a previous study performed simulations on a task-based date to indicate the monotonic relationship between GCA at the neural level and GCA in the simulated blood oxygenation level-dependent (BOLD) signals (Wen et al., 2013). However, future studies are needed to demonstrate a similar monotonic relationship in resting-state data.

## Conclusion

In conclusion, the current study found that individuals with ASD showed abnormal effective connectivity between the RTPJ and the brain regions related to the impairment of social and other domains. It helps to further elucidate the pathophysiological mechanisms of ASD and improve the early clinical screening of ASD. At the same time, it also contributes to a more directional treatment of ASD in future and promotes the development of ASD treatment.

## Data Availability Statement

Publicly available datasets were analyzed in this study. This data can be found at: http://fcon_1000.projects.nitrc.org/indi/abide/.

## Author Contributions

ZH, YS, and JS analyzed the data. ZH, YS, ML, QW, LZ, QD, XJ, and HL prepared and revised the manuscript. All authors contributed to the article and approved the submitted version.

## Funding

This work was supported by the National Natural Science Foundation of China (82001898) and the Youth Science and Technology Plan of Soochow Science and Technology Bureau and Soochow Health Planning Commission (KJXW2020065).

## Conflict of Interest

The authors declare that the research was conducted in the absence of any commercial or financial relationships that could be construed as a potential conflict of interest.

## Publisher's Note

All claims expressed in this article are solely those of the authors and do not necessarily represent those of their affiliated organizations, or those of the publisher, the editors and the reviewers. Any product that may be evaluated in this article, or claim that may be made by its manufacturer, is not guaranteed or endorsed by the publisher.

## References

[B1] Abraham A. Milham M. P. Di Martino A. Craddock R. C. Samaras D. Thirion B.. (2017). Deriving reproducible biomarkers from multi-site resting-state data: an Autism-based example. Neuroimage 147, 736–745. 10.1016/j.neuroimage.2016.10.045 27865923

[B2] Andrews-Hanna J. R. Reidler J. S. Sepulcre J. Poulin R. Buckner R. L. (2010). Functional-anatomic fractionation of the brain's default network. Neuron 65, 550–562. 10.1016/j.neuron.2010.02.005 20188659 PMC2848443

[B3] Apps M. A. Tajadura-Jiménez A. Turley G. Tsakiris M. (2012). The different faces of one's self: an fMRI study into the recognition of current and past self-facial appearances. Neuroimage 63, 1720–1729. 10.1016/j.neuroimage.2012.08.053 22940117 PMC3772343

[B4] Badillo S. Vincent T. Ciuciu P. (2013). Group-level impacts of within- and between-subject hemodynamic variability in fMRI. Neuroimage 82, 433–448. 10.1016/j.neuroimage.2013.05.100 23735261

[B5] Baumgartner T. Goette L. Guegler R. Fehr E. (2012). The mentalizing network orchestrates the impact of parochial altruism on social norm enforcement. Hum. Brain Map. 33, 1452–1469. 10.1002/hbm.21298 21574212 PMC6870290

[B6] Baxter A. J. Brugha T. S. Erskine H. E. Scheurer R. W. Vos T. Scott J. G. (2015). The epidemiology and global burden of autism spectrum disorders. Psychol. Med. 45, 601–613. 10.1017/S003329171400172X 25108395

[B7] Burnett S. Blakemore S. (2009). Functional connectivity during a social emotion task in adolescents and in adults. Eur. J. Neurosci. 29, 1294–1301. 10.1111/j.1460-9568.2009.06674.x 19302165 PMC2695858

[B8] Button K. S. Ioannidis J. P. Mokrysz C. Nosek B. A. Flint J. Robinson E. S.. (2013). Power failure: why small sample size undermines the reliability of neuroscience. Nat. Rev. Neurosci. 14, 365–376. 10.1038/nrn3475 23571845

[B9] Canigueral R. Zhang X. Noah J. A. Tachtsidis I. Hamilton A. F. d. C.. (2021). Facial and neural mechanisms during interactive disclosure of biographical information. Neuroimage 226:117572. 10.1016/j.neuroimage.2020.117572 33221448 PMC7612862

[B10] Chan M. M. Y. Han Y. M. Y. (2020). Differential mirror neuron system (MNS) activation during action observation with and without social-emotional components in autism: a meta-analysis of neuroimaging studies. Mol. Autism 11:374. 10.1186/s13229-020-00374-x 32993782 PMC7523366

[B11] Chen H. Uddin L. Q. Zhang Y. Duan X. Chen H. (2016). Atypical effective connectivity of thalamo-cortical circuits in autism spectrum disorder. Autism Res. 9, 1183–1190. 10.1002/aur.1614 27868393

[B12] Chien H. Y. Lin H. Y. Lai M. C. Gau S. S. Tseng W. Y. (2015). Hyperconnectivity of the right posterior temporo-parietal junction predicts social difficulties in boys with autism spectrum disorder. Autism Res. 8, 427–441. 10.1002/aur.1457 25630517

[B13] Committeri G. Pitzalis S. Galati G. Patria F. Pelle G. Sabatini U.. (2006). Neural bases of personal and extrapersonal neglect in humans. Brain 130, 431–441. 10.1093/brain/awl265 17008330

[B14] Craig A. D. (2009). How do you feel–now? The anterior insula and human awareness. Nat. Rev. Neurosci. 10, 59–70. 10.1038/nrn2555 19096369

[B15] Crespi B. Dinsdale N. (2019). Autism and psychosis as diametrical disorders of embodiment. Evol. Med. Public Health 2019, 121–138. 10.1093/emph/eoz021 31402979 PMC6682708

[B16] Dansereau C. Benhajali Y. Risterucci C. Pich E. M. Orban P. Arnold D.. (2017). Statistical power and prediction accuracy in multisite resting-state fMRI connectivity. Neuroimage 149, 220–232. 10.1016/j.neuroimage.2017.01.072 28161310

[B17] de la Cruz F. Wagner G. Schumann A. Suttkus S. Gullmar D. Reichenbach J. R.. (2021). Interrelations between dopamine and serotonin producing sites and regions of the default mode network. Hum. Brain Map. 42, 811–823. 10.1002/hbm.25264 33128416 PMC7814772

[B18] Di Martino A. O'Connor D. Chen B. Alaerts K. Anderson J. S. Assaf M.. (2017). Enhancing studies of the connectome in autism using the autism brain imaging data exchange II. Sci. Data 4:170010. 10.1038/sdata.2017.10 28291247 PMC5349246

[B19] Di Martino A. Yan C. G. Li Q. Denio E. Castellanos F. X. Alaerts K.. (2014). The autism brain imaging data exchange: towards a large-scale evaluation of the intrinsic brain architecture in autism. Mol. Psychiatry 19, 659–667. 10.1038/mp.2013.78 23774715 PMC4162310

[B20] Domes G. Heinrichs M. Kumbier E. Grossmann A. Hauenstein K. Herpertz S. C. (2013). Effects of intranasal oxytocin on the neural basis of face processing in autism spectrum disorder. Biol. Psychiatr. 74, 164–171. 10.1016/j.biopsych.2013.02.007 23510581

[B21] Dong C. Yang Q. Liang J. Seger C. A. Han H. Ning Y.. (2020). Impairment in the goal-directed corticostriatal learning system as a biomarker for obsessive-compulsive disorder. Psychol. Med. 50. 1490–1500. 10.1017/S0033291719001429 31272523

[B22] Fishman I. Linke A. C. Hau J. Carper R. A. Müller R. A. (2018). Atypical functional connectivity of amygdala related to reduced symptom severity in children with autism. J. Am. Acad. Child Adolesc. Psychiatry 57, 764–774. 10.1016/j.jaac.2018.06.015 30274651 PMC6230473

[B23] Fox M. D. Snyder A. Z. Vincent J. L. Corbetta M. Van Essen D. C. Raichle M. E. (2005). The human brain is intrinsically organized into dynamic, anticorrelated functional networks. Proc. Natl. Acad. Sci. U. S. A. 102, 9673–9678. 10.1073/pnas.0504136102 15976020 PMC1157105

[B24] Fox P. T. Lancaster J. L. Laird A. R. Eickhoff S. B. (2014). Meta-analysis in human neuroimaging: computational modeling of large-scale databases. Annu. Rev. Neurosci. 37, 409–434. 10.1146/annurev-neuro-062012-170320 25032500 PMC4782802

[B25] Friedman L. Stern H. Brown G. G. Mathalon D. H. Turner J. Glover G. H.. (2008). Test-retest and between-site reliability in a multicenter fMRI study. Hum. Brain Map. 29, 958–972. 10.1002/hbm.20440 17636563 PMC3670112

[B26] Friston K. J. (1994). Functional and effective connectivity in neuroimaging: a synthesis. Hum. Brain Map. 2, 56–78. 10.1002/hbm.460020107

[B27] Friston K. J. Williams S. Howard R. Frackowiak R. S. Turner R. (1996). Movement-related effects in fMRI time-series. Magn. Reson. Med. 35, 346–355. 10.1002/mrm.1910350312 8699946

[B28] Frith U. Frith C. (2010). The social brain: allowing humans to boldly go where no other species has been. Philos. Trans. R. Soc. Lond. B. Biol. Sci. 365, 165–176. 10.1098/rstb.2009.0160 20008394 PMC2842701

[B29] Goebel R. Roebroeck A. Kim D. S. Formisano E. (2003). Investigating directed cortical interactions in time-resolved fMRI data using vector autoregressive modeling and Granger causality mapping. Magn. Reson. Imaging 21, 1251–1261. 10.1016/j.mri.2003.08.026 14725933

[B30] Gotts S. J. Ramot M. Jasmin K. Martin A. (2019). Altered resting-state dynamics in autism spectrum disorder: Causal to the social impairment? Prog. Neuropsychopharmacol. Biol. Psychiatr. 90, 28–36. 10.1016/j.pnpbp.2018.11.002 30414457

[B31] Granger C. W. J. (1969). Investigating causal relations by econometric models and cross-spectral methods. Econometrica 37, 424–438. 10.2307/1912791

[B32] Griffin J. W. Bauer R. Scherf K. S. (2021). A quantitative meta-analysis of face recognition deficits in autism: 40 years of research. Psychol. Bull. 147, 268–292. 10.1037/bul0000310 33104376 PMC8961473

[B33] Hamilton J. P. Chen G. Thomason M. E. Schwartz M. E. Gotlib I. H. (2011). Investigating neural primacy in Major Depressive Disorder: multivariate Granger causality analysis of resting-state fMRI time-series data. Mol. Psychiatr. 16, 763–772. 10.1038/mp.2010.46 20479758 PMC2925061

[B34] Handwerker D. A. Gonzalez-Castillo J. D'Esposito M. Bandettini P. A. (2012). The continuing challenge of understanding and modeling hemodynamic variation in fMRI. Neuroimage 62, 1017–1023. 10.1016/j.neuroimage.2012.02.015 22366081 PMC4180210

[B35] Hickman R. A. O'Shea S. A. Mehler M. F. Chung W. K. (2022). Neurogenetic disorders across the lifespan: from aberrant development to degeneration. Nat. Rev. Neurol. 18, 117–124. 10.1038/s41582-021-00595-5 34987232 PMC10132523

[B36] Hoffmann F. Koehne S. Steinbeis N. Dziobek I. Singer T. (2016). Preserved self-other distinction during empathy in autism is linked to network integrity of right supramarginal gyrus. J. Autism. Dev. Disord. 46, 637–648. 10.1007/s10803-015-2609-0 26476740

[B37] Howlin P. Magiati I. (2017). Autism spectrum disorder: outcomes in adulthood. Curr. Opin. Psychiatr. 30, 69–76. 10.1097/YCO.0000000000000308 28067726

[B38] Humphreys K. Hasson U. Avidan G. Minshew N. Behrmann M. (2008). Cortical patterns of category-selective activation for faces, places and objects in adults with autism. Autism Res. 1, 52–63. 10.1002/aur.1 19360650 PMC2765685

[B39] Jenkinson M. Bannister P. Brady M. Smith S. (2002). Improved optimization for the robust and accurate linear registration and motion correction of brain images. NeuroImage 17, 825–841. 10.1006/nimg.2002.1132 12377157

[B40] Jia X. Z. Wang J. Sun H. Y. Zhang H. Zang Y. F. (2019). RESTplus: an improved toolkit for resting-state functional magnetic resonance imaging data processing. Sci. Bullet. 64:8. 10.1016/j.scib.2019.05.008 36659803

[B41] Jia X. Z. Zhao N. Dong H. M. Sun J. W. Barton M. Burciu R.. (2021). Small *P*-values may not yield robust findings: an example using REST-meta-PD. Sci. Bullet. 66, 2148–2152. 10.1016/j.scib.2021.06.007 36654102

[B42] Kana R. K. Libero L. E. Hu C. P. Deshpande H. D. Colburn J. S. (2014). Functional brain networks and white matter underlying theory-of-mind in autism. Soc. Cogn. Affect. Neurosci. 9, 98–105. 10.1093/scan/nss106 22977198 PMC3871731

[B43] Kana R. K. Sartin E. B. Stevens C. Jr. Deshpande H. D. Klein C. Klinger M. R.. (2017). Neural networks underlying language and social cognition during self-other processing in Autism spectrum disorders. Neuropsychologia 102, 116–123. 10.1016/j.neuropsychologia.2017.06.008 28619530

[B44] Krall S. C. Rottschy C. Oberwelland E. Bzdok D. Fox P. T. Eickhoff S. B.. (2015). The role of the right temporoparietal junction in attention and social interaction as revealed by ALE meta-analysis. Brain Struct. Funct. 220, 587–604. 10.1007/s00429-014-0803-z 24915964 PMC4791048

[B45] Kret M. E. Pichon S. Grezes J. de Gelder B. (2011). Similarities and differences in perceiving threat from dynamic faces and bodies. An fMRI study. Neuroimage 54, 1755–1762. 10.1016/j.neuroimage.2010.08.012 20723605

[B46] Li L. He C. Jian T. Guo X. Xiao J. Li Y.. (2021). Attenuated link between the medial prefrontal cortex and the amygdala in children with autism spectrum disorder: evidence from effective connectivity within the “social brain”. Prog. Neuropsychopharmacol. Biol. Psychiatr. 111:110147. 10.1016/j.pnpbp.2020.110147 33096157

[B47] Liao W. Ding J. Marinazzo D. Xu Q. Wang Z. Yuan C.. (2011). Small-world directed networks in the human brain: multivariate Granger causality analysis of resting-state fMRI. Neuroimage 54, 2683–2694. 10.1016/j.neuroimage.2010.11.007 21073960

[B48] Libero L. E. Maximo J. O. Deshpande H. D. Klinger L. G. Klinger M. R. Kana R. K. (2014). The role of mirroring and mentalizing networks in mediating action intentions in autism. Mol. Autism 5:50. 10.1186/2040-2392-5-50 25352976 PMC4210608

[B49] Lieberman M. D. (2007). Social cognitive neuroscience: a review of core processes. Ann. Rev. Psychol. 58, 259–289. 10.1146/annurev.psych.58.110405.085654 17002553

[B50] Liu P. Sutherland M. Pollick F. E. (2021). Incongruence effects in cross-modal emotional processing in autistic traits: an fMRI study. Neuropsychologia 161:107997. 10.1016/j.neuropsychologia.2021.107997 34425144

[B51] Lord C. Charman T. Havdahl A. Carbone P. Anagnostou E. Boyd B.. (2022). The Lancet Commission on the future of care and clinical research in autism. Lancet 399, 271–334. 10.1016/S0140-6736(21)01541-5 34883054

[B52] Lord C. Elsabbagh M. Baird G. Veenstra-Vanderweele J. (2018). Autism spectrum disorder. Lancet 392, 508–520. 10.1016/S0140-6736(18)31129-2 30078460 PMC7398158

[B53] Lou H. C. (2012). Paradigm shift in consciousness research: the child's self-awareness and abnormalities in autism, ADHD and schizophrenia. Acta Paediatr. 101, 112–119. 10.1111/j.1651-2227.2011.02456.x 21883452

[B54] Lowe M. J. Russell D. P. (1999). Treatment of baseline drifts in fMRI time series analysis. J. Comput. Assist. Tomogr. 23, 463–473. 10.1097/00004728-199905000-00025 10348457

[B55] Mamashli F. Kozhemiako N. Khan S. Nunes A. S. McGuiggan N. M. Losh A.. (2021). Children with autism spectrum disorder show altered functional connectivity and abnormal maturation trajectories in response to inverted faces. Autism Res. 14, 1101–1114. 10.1002/aur.2497 33709531 PMC8192491

[B56] McGonigle D. J. (2012). Test-retest reliability in fMRI: or how I learned to stop worrying and love the variability. Neuroimage 62, 1116–1120. 10.1016/j.neuroimage.2012.01.023 22261373

[B57] Miezin F. M. Maccotta L. Ollinger J. M. Petersen S. E. Buckner R. L. (2000). Characterizing the hemodynamic response: effects of presentation rate, sampling procedure, and the possibility of ordering brain activity based on relative timing. Neuroimage 11, 735–759. 10.1006/nimg.2000.0568 10860799

[B58] Müeller R. A. Fishman I. (2018). Brain connectivity and neuroimaging of social networks in autism. Trends Cogn. Sci. 22, 1103–1116. 10.1016/j.tics.2018.09.008 30391214 PMC7080636

[B59] Mul C. L. Cardini F. Stagg S. D. Sadeghi Esfahlani S. Kiourtsoglou D. Cardellicchio P.. (2019). Altered bodily self-consciousness and peripersonal space in autism. Autism 23, 2055–2067. 10.1177/1362361319838950 30943757

[B60] Müller V. I. Cieslik E. C. Laird A. R. Fox P. T. Radua J. Mataix-Cols D.. (2018). Ten simple rules for neuroimaging meta-analysis. Neurosci. Biobehav. Rev. 84, 151–161. 10.1016/j.neubiorev.2017.11.012 29180258 PMC5918306

[B61] O'Hearn K. Larsen B. Fedor J. Luna B. Lynn A. (2020). Representational similarity analysis reveals atypical age-related changes in brain regions supporting face and car recognition in autism. Neuroimage 209:116322. 10.1016/j.neuroimage.2019.116322 31786166

[B62] Olivé I. Tempelmann C. Berthoz A. Heinze H. J. (2015). Increased functional connectivity between superior colliculus and brain regions implicated in bodily self-consciousness during the rubber hand illusion. Hum. Brain Map. 36, 717–730. 10.1002/hbm.22659 25346407 PMC6869389

[B63] Park H. D. Blanke O. (2019). Coupling inner and outer body for self-consciousness. Trends Cogn Sci. 23, 377–388. 10.1016/j.tics.2019.02.002 30826212

[B64] Patel G. H. Sestieri C. Corbetta M. (2019). The evolution of the temporoparietal junction and posterior superior temporal sulcus. Cortex 118, 38–50. 10.1016/j.cortex.2019.01.026 30808550 PMC7713666

[B65] Pelphrey K. A. Shultz S. Hudac C. M. Wyk B. C. V. (2011). Research Review: constraining heterogeneity: the social brain and its development in autism spectrum disorder. J. Child Psychol. Psychiatr. 52, 631–644. 10.1111/j.1469-7610.2010.02349.x 21244421 PMC3096715

[B66] Poon K. K. Sidhu D. J. (2017). Adults with autism spectrum disorders: a review of outcomes, social attainment, and interventions. Curr. Opin. Psychiatry. 30, 77–84. 10.1097/YCO.0000000000000306 28009723

[B67] Price C. J. Devlin J. T. (2011). The Interactive Account of ventral occipitotemporal contributions to reading. Trends Cogn. Sci. 15, 246–253. 10.1016/j.tics.2011.04.001 21549634 PMC3223525

[B68] Qin P. Wang M. Northoff G. (2020). Linking bodily, environmental and mental states in the self-a three-level model based on a meta-analysis. Neurosci. Biobehav. Rev. 115, 77–95. 10.1016/j.neubiorev.2020.05.004 32492474

[B69] Rabellino D. Densmore M. Théberge J. McKinnon M. C. Lanius R. A. (2018). The cerebellum after trauma: resting-state functional connectivity of the cerebellum in posttraumatic stress disorder and its dissociative subtype. Hum. Brain Map. 39, 3354–3374. 10.1002/hbm.24081 29667267 PMC6866303

[B70] Radua J. Mataix-Cols D. Phillips M. L. El-Hage W. Kronhaus D. M. Cardoner N.. (2012). A new meta-analytic method for neuroimaging studies that combines reported peak coordinates and statistical parametric maps. Eur. Psychiatr. 27, 605–611. 10.1016/j.eurpsy.2011.04.001 21658917

[B71] Radua J. Rubia K. Canales-Rodríguez E. J. Pomarol-Clotet E. Fusar-Poli P. Mataix-Cols D. (2014). Anisotropic kernels for coordinate-based meta-analyses of neuroimaging studies. Front. Psychiatr. 5:13. 10.3389/fpsyt.2014.00013 24575054 PMC3919071

[B72] Rangaprakash D. Wu G.-R. Marinazzo D. Hu X. Deshpande G. (2018). Hemodynamic response function (HRF) variability confounds resting-state fMRI functional connectivity. Magn. Reson. Med. 80, 1697–1713. 10.1002/mrm.27146 29656446

[B73] Roebroeck A. Formisano E. Goebel R. (2005). Mapping directed influence over the brain using Granger causality and fMRI. Neuroimage 25, 230–242. 10.1016/j.neuroimage.2004.11.017 15734358

[B74] Rolls E. T. Zhou Y. Cheng W. Gilson M. Deco G. Feng J. (2020). Effective connectivity in autism. Autism Res. 13, 32-44. 10.1002/aur.2235 31657138

[B75] Salimi-Khorshidi G. Smith S. M. Keltner J. R. Wager T. D. Nichols T. E. (2009). Meta-analysis of neuroimaging data: a comparison of image-based and coordinate-based pooling of studies. Neuroimage 45, 810–823. 10.1016/j.neuroimage.2008.12.039 19166944

[B76] Saur D. Kreher B. W. Schnell S. Kümmerer D. Kellmeyer P. Vry M. S.. (2008). Ventral and dorsal pathways for language. Proc. Natl. Acad. Sci. U. S. A. 105, 18035–18040. 10.1073/pnas.0805234105 19004769 PMC2584675

[B77] Sharma S. R. Gonda X. Tarazi F. I. (2018). Autism Spectrum Disorder: classification, diagnosis and therapy. Pharmacol. Therapeut. 190, 91–104. 10.1016/j.pharmthera.2018.05.007 29763648

[B78] Shi Y. Liu W. Liu R. Zeng Y. Wu L. Huang S.. (2019). Investigation of the emotional network in depression after stroke: a study of multivariate Granger causality analysis of fMRI data. J. Affect. Disord. 249, 35–44. 10.1016/j.jad.2019.02.020 30743020

[B79] Tang H. Mai X. Wang S. Zhu C. Krueger F. Liu C. (2016). Interpersonal brain synchronization in the right temporo-parietal junction during face-to-face economic exchange. Soc. Cogn. Affect. Neurosci. 11, 23–32. 10.1093/scan/nsv092 26211014 PMC4692317

[B80] Turner R. (1997). Signal sources in bold contrast fMRI. Adv. Exp. Med. Biol. 413, 19–25. 10.1007/978-1-4899-0056-2_2 9238481

[B81] Uddin L. Q. Menon V. Young C. B. Ryali S. Chen T. Khouzam A.. (2011). Multivariate searchlight classification of structural magnetic resonance imaging in children and adolescents with autism. Biol. Psychiatr. 70, 833–841. 10.1016/j.biopsych.2011.07.014 21890111 PMC3191298

[B82] Van Horn J. D. Toga A. W. (2009). Multisite neuroimaging trials. Curr. Opin. Neurol. 22, 370–378. 10.1097/WCO.0b013e32832d92de 19506479 PMC2777976

[B83] van Veluw S. J. Chance S. A. (2014). Differentiating between self and others: an ALE meta-analysis of fMRI studies of self-recognition and theory of mind. Brain Imaging Behav. 8, 24–38. 10.1007/s11682-013-9266-8 24535033

[B84] Verhoeven E. W. Marijnissen N. Berger H. J. Oudshoorn J. van der Sijde A. Teunisse J. P. (2012). Brief report: relationship between self-awareness of real-world behavior and treatment outcome in autism spectrum disorders. J. Autism Dev. Disord. 42, 889–894. 10.1007/s10803-011-1311-0 21698498

[B85] Wang L. Wei Q. Wang C. Xu J. Wang K. Tian Y.. (2020a). Altered functional connectivity patterns of insular subregions in major depressive disorder after electroconvulsive therapy. Brain Imaging Behav. 14, 753–761. 10.1007/s11682-018-0013-z 30610527

[B86] Wang M. Zeng N. Zheng H. Du X. Potenza M. N. Dong G. H. (2022). Altered effective connectivity from the pregenual anterior cingulate cortex to the laterobasal amygdala mediates the relationship between internet gaming disorder and loneliness. Psychol. Med. 52, 737–746. 10.1017/S0033291720002366 32684185

[B87] Wang S. Tepfer L. J. Taren A. A. Smith D. V. (2020b). Functional parcellation of the default mode network: a large-scale meta-analysis. Sci. Rep. 10:8. 10.1038/s41598-020-72317-8 32999307 PMC7528067

[B88] Wei H. L. Chen J. Chen Y.-C. Yu Y.-S. Guo X. Zhou G.-P.. (2020). Impaired effective functional connectivity of the sensorimotor network in interictal episodic migraineurs without aura. J. Headache Pain 21:5. 10.1186/s10194-020-01176-5 32928098 PMC7489040

[B89] Wei M. Qin J. Yan R. Bi K. Liu C. Yao Z.. (2015). Association of resting-state network dysfunction with their dynamics of inter-network interactions in depression. J. Affect. Disord. 174, 527–534. 10.1016/j.jad.2014.12.020 25556670

[B90] Wen X. Rangarajan G. Ding M. (2013). Is Granger causality a viable technique for analyzing fMRI data? PLoS ONE 8:e67428. 10.1371/journal.pone.0067428 23861763 PMC3701552

[B91] Wicker B. Fonlupt P. Hubert B. Tardif C. Gepner B. Deruelle C. (2008). Abnormal cerebral effective connectivity during explicit emotional processing in adults with autism spectrum disorder. Soc. Cogn. Affect. Neurosci. 3, 135–143. 10.1093/scan/nsn007 19015104 PMC2555468

[B92] Wiebking C. de Greck M. Duncan N. W. Tempelmann C. Bajbouj M. Northoff G. (2015). Interoception in insula subregions as a possible state marker for depression - an exploratory fMRI study investigating healthy, depressed and remitted participants. Front. Behav. Neurosci. 9:82. 10.3389/fnbeh.2015.00082 25914633 PMC4392695

[B93] Wiebking C. Duncan N. W. Tiret B. Hayes D. J. Marjanska M. Doyon J.. (2014). GABA in the insula - a predictor of the neural response to interoceptive awareness. Neuroimage 86, 10–18. 10.1016/j.neuroimage.2013.04.042 23618604 PMC4386686

[B94] Wood J. J. Kendall P. C. Wood K. S. Kerns C. M. Seltzer M. Small B. J.. (2020). Cognitive behavioral treatments for anxiety in children with autism spectrum disorder a randomized clinical trial. J Am. Med. Assoc. Psychiatr. 77, 474–483. 10.1001/jamapsychiatry.2019.4160 31755906 PMC6902190

[B95] Woodward N. D. Giraldo-Chica M. Rogers B. Cascio C. J. (2017). Thalamocortical dysconnectivity in autism spectrum disorder: an analysis of the Autism Brain Imaging Data Exchange. Biol. Psychiatr. Cogn. Neurosci. Neuroimag. 2, 76–84. 10.1016/j.bpsc.2016.09.002 28584881 PMC5455796

[B96] Wu G.-R. Di Perri C. Charland-Verville V. Martial C. Carriere M. Vanhaudenhuyse A.. (2019). Modulation of the spontaneous hemodynamic response function across levels of consciousness. Neuroimage 200, 450–459. 10.1016/j.neuroimage.2019.07.011 31284028

[B97] Wu G.-R. Marinazzo D. (2015). Point-process deconvolution of fMRI BOLD signal reveals effective connectivity alterations in chronic pain patients. Brain Topogr. 28, 541–547. 10.1007/s10548-014-0404-4 25281022

[B98] Wu G.-R. Marinazzo D. (2016). Sensitivity of the resting-state haemodynamic response function estimation to autonomic nervous system fluctuations. Philos. Trans. A Math. Phys. Eng. Sci. 374:190. 10.1098/rsta.2015.0190 27044997 PMC4822449

[B99] Wu G. R. Colenbier N. Van Den Bossche S. Clauw K. Johri A. Tandon M.. (2021). rsHRF: a toolbox for resting-state HRF estimation and deconvolution. Neuroimage 244:118591. 10.1016/j.neuroimage.2021.118591 34560269

[B100] Wu G. R. Liao W. Stramaglia S. Ding J. R. Chen H. Marinazzo D. (2013). A blind deconvolution approach to recover effective connectivity brain networks from resting state fMRI data. Med. Image Anal. 17, 365–374. 10.1016/j.media.2013.01.003 23422254

[B101] Xu G. Strathearn L. Liu B. O'Brien M. Kopelman T. G. Zhu J.. (2019). Prevalence and treatment patterns of autism spectrum disorder in the United States, 2016. J. Am. Med. Assoc. Pediatr. 173, 153–159. 10.1001/jamapediatrics.2018.4208 30508021 PMC6439607

[B102] Yan C. G. Zang Y. F. (2010). DPARSF: a MATLAB toolbox for “pipeline” data analysis of resting-state fMRI. Front. Syst. Neurosci. 4, 13–13. 10.3389/fnsys.2010.00013 20577591 PMC2889691

[B103] Yu M. Linn K. A. Cook P. A. Phillips M. L. Mcinnis M. Fava M.. (2018). Statistical harmonization corrects site effects in functional connectivity measurements from multi-site fMRI data. Hum. Brain Map. 39, 4213–4227. 10.1002/hbm.24241 29962049 PMC6179920

[B104] Zang Z. X. Yan C. G. Dong Z. Y. Huang J. Zang Y. F. (2012). Granger causality analysis implementation on MATLAB: a graphic user interface toolkit for fMRI data processing. J. Neurosci. Methods. 203, 418–426. 10.1016/j.jneumeth.2011.10.006 22020117

[B105] Zhao L. Xue S. W. Sun Y. K. Lan Z. Zhang Z. Xue Y.. (2022). Altered dynamic functional connectivity of insular subregions could predict symptom severity of male patients with autism spectrum disorder. J. Affect. Disord. 299, 504–512. 10.1016/j.jad.2021.12.093 34953921

