# Supplementary material for: The Atypical Effective Connectivity of Right Temporoparietal Junction in Autism Spectrum Disorder: A Multi-Site Study
Source: Front Neurosci. 2022 Jul 18;16:927556. doi: 10.3389/fnins.2022.927556 (PMC9340667; doi:10.3389/fnins.2022.927556)
Supplement: Supplementary file 1 [file Table_1.docx]

|  | **Table S1** Image Quality Statistics Table | | | | | | | | | |
| --- | --- | --- | --- | --- | --- | --- | --- | --- | --- | --- |
| Site ID | | TR (ms) | Slice | Timepoint | Total Sub (ASD/TD) | Included Sub  (ASD/TD) | Image Quality | Normalize | Motion | Handedness |
| 001_BNI | | 3000 | 50 | 119 | 29/29 | 22/21 | 1 | 14 |  |  |
| 002_Caltech | | 2000 | 34 | 150 | 19/19 | 4/10 | 1 | 23 |  |  |
| 003_EMC | | 2000 | 37 | 160 | 27/27 | 13/14 | 1 | 5 | 14 | 7 |
| 004_ETH | | 2000 | 40 | 210 | 13/24 | 6/19 | 1 | 1 | 10 |  |
| 005_GU | | 2000 | 43 | 152 | 51/55 | 22/31 |  | 35 | 11 | 7 |
| 006_IP | | 2700 | 32 | 85 | 22/34 | 15/17 | 1 | 14 | 3 | 6 |
| 007_IU | | 813 | 42 | 433 | 20/20 | 12/15 | 1 | 7 |  | 5 |
| 008_KKI_1 | | 2500 | 47 | 121 | 20/38 | 13/28 | 2 | 1 | 6 | 8 |
| 009_KKI_2 | | 2500 | 47 | 156 | 58/150 | 41/108 |  | 5 | 36 | 16 |
| 010_Leuven | | 1667 | 32 | 250 | 29/35 | 24/27 |  | 11 | 2 |  |
| 011_MaxMun_1 | | 3000 | 28 | 120 | 11/12 | 11/12 | 1 |  |  |  |
| 012_MaxMun_2 | | 3000 | 40 | 120 | 2/15 | 2/14 |  |  | 1 |  |
| 013_MaxMun_3 | | 3000 | 40 | 200 | 10/6 | 8/2 |  |  | 6 |  |
| 014_NYU | | 2000 | 33 | 172 | 127/135 | 67/98 |  | 4 | 10 | 83 |
| 015_OHSU_1 | | 2500 | 36 | 82 | 13/15 | 13/13 |  |  | 2 |  |
| 016_OHSU_2 | | 2500 | 36 | 120 | 37/56 | 31/51 |  | 4 | 5 | 2 |
| 017_Olin1 | | 1500 | 29 | 210 | 20/16 | 14/11 |  | 3 | 8 |  |
| 018_Olin2 | | 475 | 48 | 932 | 24/35 | 10/29 |  | 3 | 9 | 8 |
| 019_Pitt | | 1500 | 29 | 200 | 30/27 | 22/23 |  | 9 | 3 |  |
| 020_SBL | | 2200 | 38 | 200 | 15/15 | 9/10 |  | 5 | 1 | 5 |
| 021_SDSU | | 2000 | 42 | 180 | 47/47 | 37/41 |  |  | 5 | 11 |

| 022_Stanford1 | | 2000 | 29 | 180 | 20/20 | 17/16 | 1 | 2 | 4 |  |
| --- | --- | --- | --- | --- | --- | --- | --- | --- | --- | --- |
| 023_Stanford2 | | 2000 | 31 | 180 | 21/21 | 17/18 | 1 |  | 4 | 2 |
| 024_Trinity1 | | 2000 | 38 | 150 | 24/25 | 20/23 | 1 |  | 5 |  |
| 025_Trinity2 | | 2000 | 37 | 210 | 21/21 | 13/18 | 1 | 1 | 9 |  |
| 026_UMIA | | 2000 | 42 | 295 | 13/15 | 10/8 | 3 | 1 | 6 |  |
| 027_UCD | | 2000 | 36 | 151 | 18/14 | 16/13 |  | 1 | 1 | 1 |
| 028_UCLA | | 3000 | 34 | 120 | 78/63 | 49/47 | 10 | 18 | 14 | 3 |
| 029_UM | | 2000 | 40 | 300 | 68/77 | 37/59 |  | 18 | 26 | 5 |
| 030_USM | | 2000 | 40 | 236 | 75/59 | 43/40 | 4 | 1 | 27 | 19 |
|  | *Note:* The numbers of Total Sub and Included Sub represent the numbers of ASD and the numbers of TD.  The *003_CMU1* (n=14) and *004_CMU2* (n=13) were excluded due to their poor standardization. Because of no TD participants, the *012_KUL* (n=28) and *018_NYU2* (n=27) were excluded. And the *035_YALE* site was excluded due to it couldn’t complete the preprocessing. | | | | | | | | | |
